# Analysis of quality of life and physical activity in a sample of schoolchildren and adolescents with attention deficit hyperactivity disorder

**DOI:** 10.1192/j.eurpsy.2026.10555

**Published:** 2026-07-31

**Authors:** S. Pérez-Sánchez, C. Pérez-Sánchez, E. Gonzalez-Reigadas

**Affiliations:** 1 Psychiatry; 2 Medicina Familia, Servicio Murciano de Salud, Murcia, Spain

## Abstract

**Introduction:**

Attention Deficit Hyperactivity Disorder (ADHD) is a condition that leads to significant difficulties in functional living and is associated with excessive screen use, behavioral disturbances, and sleep disorders. Studies indicate that engaging in structured physical activity (PA) and quality of life (QoL) during childhood and adolescence may have a positive influence on cognitive and socio-emotional development.

**Keywords/ Descriptors:**

ADHD, Quality of Life, Physical Activity, Childhood and Adolescence

**Objectives:**

To analyze the quality of life in a pediatric ADHD sample that engages in PA and to examine whether there are differences compared to those who do not engage in PA.

**Methods:**

A cross-sectional, descriptive, and observational study was conducted with a clinical sample of 82 children and adolescents diagnosed with ADHD. Data were analyzed descriptively and stratified, and possible associations between variables were examined.

**Results:**

Of the sample, 30.49% engaged in PA. Physical activity was significantly associated with better quality of life (QoL), showing a strong positive correlation (r = .74; p < .01). Multiple regression analysis identified additional predictors of better QoL.

**Conclusions:**

In this study, only one-third of ADHD participants engaged in PA and showed fewer comorbidities than those who did not. Furthermore, this group reported a better perception of their QoL. These findings support the need for longitudinal and interventional studies to evaluate the sustained and specific effects of different types of PA on QoL in this population.

**Disclosure of Interest:**

None Declared

